# Retrograde procedural memory is impaired in people with Parkinson’s disease with freezing of gait

**DOI:** 10.3389/fnagi.2023.1296323

**Published:** 2024-01-05

**Authors:** Laure Pauly, Claire Pauly, Maxime Hansen, Valerie E. Schröder, Armin Rauschenberger, Anja K. Leist, Rejko Krüger

**Affiliations:** ^1^Transversal Translational Medicine, Luxembourg Institute of Health, Strassen, Luxembourg; ^2^Faculty of Science, Technology and Medicine, University of Luxembourg, Esch-sur-Alzette, Luxembourg; ^3^Luxembourg Centre for Systems Biomedicine, University of Luxembourg, Esch-sur-Alzette, Luxembourg; ^4^Parkinson Research Clinic, Centre Hospitalier de Luxembourg, Strassen, Luxembourg; ^5^Department of Social Sciences, Institute for Research on Socio-Economic Inequality, University of Luxembourg, Esch-sur-Alzette, Luxembourg

**Keywords:** freezing of gait, Parkinson’s disease, cognitive impairment, procedural memory, gait impairment

## Abstract

**Background:**

Freezing of gait (FOG), is associated with impairment of different cognitive functions. Previous studies hypothesized that FOG may be due to a loss of automaticity.

**Research question:**

To explore whether FOG is associated with impairment in cognitive functions, focusing on retrograde procedural memory, the memory responsible for the automatic, implicit stored procedures that have been acquired in earlier life stages.

**Methods:**

In this cross-sectional, case–control study, 288 people with typical Parkinson’s disease (PD) from the Luxembourg Parkinson’s Study were assigned to Freezers (FOG^+^) and non-Freezers (FOG^−^) based on the MDS-UPDRS 2.13 (self-reported FOG episodes) and 3.11 (FOG evaluated by clinicians during gait assessment). Both groups were matched on age, sex and disease duration. Global cognition (MoCA), retrograde procedural memory and visuo-constructive abilities (CUPRO), psychomotor speed and mental flexibility (TMT) were assessed. Furthermore, we repeated our analyses by additionally controlling for depression (BDI-I).

**Results:**

Besides lower global cognition (MoCA; *p* = 0.007) and mental flexibility (TMT-B and Delta-TMT; *p* < 0.001), FOG^+^ showed a lower performance in retrograde procedural memory (CUPRO-IS1; *p* < 0.001) compared to FOG^−^. After controlling additionally for depression, our main outcome variable CUPRO-IS1 remained significantly lower in FOG^+^ (*p* = 0.010).

**Conclusion:**

Our findings demonstrated that besides lower global cognition and mental flexibility scores, FOG^+^ showed lower performance in retrograde procedural memory compared to matched FOG-control patients, even when accounting for factors such as age, sex, disease duration or depression.

**Significance:**

In the context of limited treatment options, especially for non-invasive therapeutic approaches, these insights on procedural memory and FOG may lead to new hypotheses on FOG etiology and consequently the development of new treatment options.

## 1 Introduction

Freezing of gait (FOG) is an abnormal gait pattern, defined by brief, temporary episodes of difficulty or even inability to move the feet despite the intention to walk (Nutt et al., 2011). FOG is common in people with Parkinson’s disease (PD), affecting approximately 38% in early disease stages and up to 65% in advanced disease stages (Zhang et al., 2021). By reducing quality of life and independence, FOG poses a substantial burden for patients and caregivers (Perez-Lloret et al., 2014). Besides a proven link with disease progression (Macht et al., 2007), additional symptoms like impaired cognition are observed in People with PD (PwPD) with FOG (FOG^+^) (Peterson et al., 2016).

Early findings indicated that gait is controlled by the central pattern generators in the spinal cord and brain stem. Even though these brain areas are highly implicated in locomotion, recent evidence from behavioral and imaging studies demonstrated the implication of higher-level cortical structures in gait, highlighting the importance of cognitive function in the process (Peterson et al., 2016). Nevertheless, studies related to this topic are still controversial. Some studies suggested impaired cognition, most notably in executive and visuospatial functions in FOG^+^ (Amboni et al., 2007; Cohen et al., 2014; Jha et al., 2015; Peterson et al., 2016; Gan et al., 2023). Others provided no evidence for differences in cognition (Morris et al., 2020; Taximaimaiti and Wang, 2021). These divergences could be due to the heterogeneity in their study populations (e.g., age, education), covariates (e.g., disease severity and medication), varying definitions of FOG, different applied neuropsychological assessments or to the fact that cognitive functions more directly associated with FOG have not been tested yet.

Despite that FOG in PD has been characterized as a de-automatization deficit (Hallett, 2008; Nutt et al., 2011; Heremans et al., 2013; Vandenbossche et al., 2013b), little is known about the relation between FOG and procedural memory. In PD, movements become less automatic, mainly due to the loss of dopaminergic input to the striatum, a brain area that plays an essential role in procedural skills, such as walking (Lehéricy et al., 2005; Doyon et al., 2009). Therefore, these skills require more attentional control in PD, relying on a shift in neural activation from sub-cortical, implicit and automatic behaviour to cortical brain areas, explicit and goal-directed behavior, as a compensation strategy (Vandenbossche et al., 2013b; Wu et al., 2015).

As procedural memory is often imprecisely defined, and we have judged Crystal’s and colleagues’ definition, which divides procedural memory into anterograde and retrograde components, as the most accurate, we are applying this terminology in our study (Crystal et al., 1989). Anterograde procedural memory involves the acquisition of new skills, while the ability to execute skills acquired in earlier life stages is part of retrograde procedural memory. An affected anterograde procedural memory demonstrates difficulties with the ability to learn new skills, by repetition. An affected retrograde procedural memory demonstrates difficulties in recalling and executing learned procedural skills that had once reached automatization. Despite numerous studies on procedural memory in PD, the conclusions are inconsistent. Studies on procedural memory in PD investigated mainly the anterograde procedural memory. While most of these studies have described an impairment of the learning of new procedural skills (Frith et al., 1986; Saint-Cyr et al., 1988; Heindel et al., 1989; Ferraro et al., 1993; Jackson et al., 1995; Roncacci et al., 1996; Westwater et al., 1998; Krebs et al., 2001; Sarazin et al., 2002; Muslimović et al., 2007; Vandenbossche et al., 2013a; Vakil et al., 2021) others did not observe any significant differences (Agostino, 1996; Seidler et al., 2007; Beauchamp et al., 2008; Pendt et al., 2011; Panouillères et al., 2016) or only mild to moderate or partial impairments (Harrington et al., 1990; Pascual-Leone et al., 1993; Allain et al., 1995; Thomas-Antérion et al., 1996; Sommer et al., 1999). Even though, the unique characteristic of procedural memory is its robustness in time, and its longevity, only a limited number of studies investigated retrograde procedural memory (Mochizuki-Kawai et al., 2004; Cohen and Pourcher, 2007; Heremans et al., 2016). Assessing procedural memory still encounters difficulties on both levels, theoretical and clinical (Van der Linden and Seron, 2014). This might be explained by the not fully understood theoretical background and the lack of easy-to-apply assessments. Specific impairments in procedural skills, like handwriting (Heremans et al., 2016), and in the acquiring new procedural skills (Vandenbossche et al., 2013a) in FOG^+^ compared to a control group without FOG (FOG^−^), have been documented. However, to our knowledge, apart from the previously mentioned handwriting studies, retrograde procedural memory has not been systematically assessed in FOG^+^. We recently provided the extended evaluation system of the Cube Copying Task, the CUPRO (short for CUbe drawing PROcedure) to assess this memory concept and demonstrated impairments in PwPD compared to matched control subjects (Pauly et al., 2022). We hypothesized that the Cube Copying Task meets the conditions of assessing retrograde procedural memory: by copying the Cube, a (i) previously acquireded procedureis (ii) unconciously applied. Through assessing discriminant validity with several tests representing related constructs, no evidence was found indicating that general motor symptoms prevalent in PD as well as deficits in visuo-cognition, planning or executive functions might interfere with the Cube coping performance (Pauly et al., 2022). Aiming to investigate the relationship between cognition and FOG, with a focus on retrograde procedural memory, we hypothesized that this memory deficit, already observed in PD (Pauly et al., 2022), may be more prominent among FOG^+^ compared to matched FOG^−^. In addition to procedural memory, we assessed global cognition, visuo-constructive function, psychomotor speed and mental flexibility.

Despite that FOG is one of the main causes of falls and reduced quality of life, knowledge of treatment options, especially for non-invasive therapeutic approaches, is limited (Perez-Lloret et al., 2014; Walton et al., 2015). Previous studies demonstrated improved FOG symptoms in PwPD after cognitive rehabilitative training that may lead to neuroplastic changes by reinforcing cognitive strategies (Walton et al., 2018). Therefore, insights on specific cognitive impairment patterns, such as on procedural memory in PwPD and FOG may lead to a better understanding of the etiology of FOG. Consequently, a better characterization of the cognitive impairments observed in PD may support the targeted development of cognitive rehabilitation training and reeducation therapies, aiming to maintain, or even reinforce cognitive function and indirectly improve the quality of life of PwPD.

## 2 Materials and methods

### 2.1 Participants

Participants were recruited from the Luxembourg Parkinson’s Study of the National Centre of Excellence in Research on PD (NCER-PD) (Hipp et al., 2018) and provided informed consent according to the Declaration of Helsinki. Inclusion criteria were the age of 18 years or older and the ability to sign the consent. We excluded participants with Deep Brain Stimulation, with a Montreal Cognitive Assessment (MoCA) score below 21 or having been diagnosed with PD with dementia (Dubois et al., 2007), atypical forms of parkinsonism, other neurological or severe psychiatric disorders. In the present study, 288 participants with typical PD were selected and two groups were defined differing in FOG status (FOG^+^ = 144; FOG^−^ = 144) propensity score matched on age, sex and disease duration. The diagnosis was based on the UK PD Society Brain Bank Clinical Diagnostic Criteria (Hughes et al., 1992). Each subject underwent a neurological examination and provided information on early symptoms, disease history and current treatment. Patients were tested while being on their regular medication. Levodopa Equivalent Daily Dose (LEDD) was calculated for each participant (Tomlinson et al., 2010). The Movement Disorder Society - Unified Parkinson’s Disease Rating Scale (MDS-UPDRS) (Goetz et al., 2007) and the modified Hoehn and Yahr scale (Goetz et al., 2004) were used to assess motor symptoms and disease stages.

### 2.2 FOG evaluation

Current and past FOG symptoms were explored using information (i) on self-reported FOG episodes (MDS-UPDRS part II sub-item 2.13), and (ii) on FOG symptoms reported by the specialized neurologist during gait assessment (MDS-UPDRS part III sub-item 3.11). Participants were categorized into two groups; the FOG^+^ group with participants reporting or presenting FOG episodes (MDS-UPDRS 2.13 or MDS-UPDRS 3.11 score range 1–4) in at least one of their visits at the research clinic and the FOG-group without FOG symptoms (MDS-UPDRS 2.13 = 0 and MDS-UPDRS 3.11 = 0).

A detailed flowchart can be found in the Supplementary Figure S1.

### 2.3 Neuropsychological assessment

With the CUPRO evaluation system, we assessed our main outcome variable, the Cube copying procedure (Intermediate Score 1 – IS1), representing retrograde procedural memory and the final result of the Cube (Intermediate Score 2 – IS2), representing visuo-constructive functions (Pauly et al., 2022).

The CUPRO is an extended evaluation score for the Cube Copying Task, that was initially evaluated, with the classical scoring system established by Nasreddine et al. (2005). Following the classical scoring system, one point was administered for a correct final result, assessing visuo-constructive functions: the drawing must be three-dimensional, the orientation of the drawing must be correct, the final result must be correct and the point was not given if any of these criteria were not met. We extended upon this scoring system to separately assess its three-dimensionality (1 point), the accuracy of its orientation (1 point), and the correctness of the final result (1 point) [Intermediate Score 2 (IS_2_)]. Subsequently, the Cube Copying Task was further extended to additionally evaluate the copying procedure itself. Based on the four, previously defined typical procedures, the extended scoring system evaluates the starting approach; 1 point is given if the subject started with one of the squares, surfaces, or the three axes. Further, the procedure itself is evaluated on 1 point (A.-D.). The final point is administered if the subject completes the copying procedure, by connecting the lines [Intermediate Score1 (IS_1_)]. To summarize, the total score of six points of the CUPRO evaluation system is composed of two intermediate scores. The first intermediate score of three points (IS_1_) evaluates the copying procedure. The second intermediate score (IS_2_) of three points allows us to infer aspects related to visuo-constructive functions. A detailed description of the development of the CUPRO can be found elsewhere (Pauly et al., 2022).

In addition to retrograde procedural memory, global cognition was evaluated with the MoCA (Nasreddine et al., 2005). Psychomotor speed and mental flexibility were measured with the Trail-Making-Test (TMT) part A and part B, respectively (Godefroy, 2008). Delta TMT is defined as (TMT-B) – (TMT-A).

### 2.4 Questionnaires

The Beck Depression Inventory (BDI-I) (Beck et al., 1961), Starkstein Apathy Scale (SAS) (Starkstein et al., 1992), PD Questionnaire (PDQ-39) (Peto et al., 1995), and the MDS-UPDRS (I&II) (Goetz et al., 2007), were used to assess the presence of depression symptomatology, apathy, quality of life, and non-motor and motor aspects of experiences of daily living, respectively. Functional Activity Questionnaire (FAQ) (Pfeffer et al., 1982) and the Short Informant Questionnaire on Cognitive Decline in the Elderly (IQCODE) (Jorm, 1994) were used to measure functional activity and cognitive decline reported by relatives.

For all the questionnaires, we investigate the summed item scores, except for the PDQ-39 we investigate the summary index, derived by the sum of all 39 items’ responses as a percentage score (Jenkinson et al., 1997).

### 2.5 Genetical testing

Targeted Glucocerebrosidase (GBA) screening was performed by real-time single-molecule sequencing developed by Pacific BioScience (PacBio). More details about the GBA screening have been reported elsewhere (Pachchek et al., 2023). In the present study, we define the carriers of a known pathogenic mutation in the GBA gene as GBA^+^ and the non-carriers as GBA^−^.

### 2.6 Statistics

The two groups were matched by age, sex and disease duration by propensity score matching (matching tolerance = 0.05). Differences in demographic and clinical characteristics as well as cognitive performance were analyzed using the non-parametric Mann–Whitney U test and Pearson’s chi-squared test (two-tailed). Correlations were tested with the bivariate Spearman correlation test. The significance threshold was set at *p* ≤ 0.05. Where appropriate, we used the Bonferroni correction for multiple testing to prevent alpha error inflation. The same statistical analyses were repeated for the two groups matched additionally for depression (Supplementary material). All statistical analyses were performed using R version 4.2.0 GUI 1.78 and RStudio version 2023.03.1 + 446.

## 3 Results

Testing for sociodemographic differences between the groups confirmed successful matching, as the groups did not differ significantly in sex [χ^2^(1, *N* = 288) = 0.06, *p* = 0.802], age (*u* = 10,379, *p* = 0.988), or disease duration (*u* = 11,740, *p* = 0.677). After multiple testing corrections, FOG^+^ presented significantly higher scores for MDS-UPDRS-I (*u* = 6116.5, *p* < 0.001), II (*u* = 4955.5, *p* < 0.001) and III (*u* = 7853.5, *p* < 0.001), BDI-I (*u* = 6,418, *p* < 0.001), SAS (*u* = 6,627, *p* < 0.001), FAQ (*u* = 5,483, *p* < 0.001) and PDQ-39 (*u* = 4,820, *p* < 0.001). FOG^+^ presented nominally significantly higher scores for the short IQCODE (*u* = 6564.5, *p* = 0.039). FOG^+^ present shorter education duration and higher LEDD than FOG^−^, but these differences are insignificant (*u* = 11,740, *p* = 0.051; *u* = 7,304, *p* = 0.059 respectively). Similarly, the number of languages is not significantly different. Given that heterozygous GBA gene mutation carriers (severe, mild and low-risk pathogenic mutations) represent increased susceptibility for PD, gait impairment and cognitive dysfunction (Wang et al., 2014), we tested group differences. No significant difference was observed regarding the number of GBA carriers between the groups [*χ*^2^(1, *N* = 259) = 0.89, *p* = 0.583]. Descriptive statistics on the demographic and clinical data can be found in Table 1.

**Table 1:** Demographic and clinical data for FOG+ (*N* = 144) and FOG- (*N* = 144).

Variable	Descriptive statistics		*p*-values	Significance
	FOG^+^	FOG^−^	FOG+ vs. FOG-	
**N Total**	144	144		
**N GBA+/GBA-**	19/119^+6NA^	13/108^+23NA^	*p* = 0.583	
**Sex, M/F**	95/49	98/46	*p* = 0.802	

	*Mean*	*SD*	*Median*	*IQR*	*N*	*Mean*	*SD*	*Median*	*IQR*	*N*		
**Age**, _**in years**_	66.94	10.11	68.48	14.83	144	67.51	9.21	68.55	12.70	144	*p* = 0.988	
**Disease duration**, _**in years**_	4.67	4.03	4.00	4.00	144	4.50	3.99	3.50	4.00	144	*p* = 0.677	
**Education**, _**in years**_	12.99	3.98	12.00	4.25	144	13.87	3.80	13.00	6.00	144	*p* = 0.051	
**Languages spoken**	2.90	1.08	3.00	2.00	140	2.81	1.10	3.00	2.00	140	*p* = 0.527	
**MDS-UPDRS I (/52)**	11.55	6.38	11.00	8.00	141	7.71	4.91	7.00	6.00	138	*p* < 0.001	**
**MDS-UPDRS II (/52)**	12.99	7.30	12.00	9.00	141	7.25	4.82	6.00	5.50	139	*p* < 0.001	**
**MDS-UPDRS III (/132)**	37.80	12.98	36.00	16.75	142	32.24	12.64	32.00	17.25	144	*p* < 0.001	**
**Modified Hoehn and Yahr**	2.27	0.55	2.00	0.50	143	1.98	0.42	2.00	0.00	144	*p* < 0.001	**
Stage 1/1.5/2/2.5/3/4/5	2/4/88/26/16/7/0^+1NA^					11/12/99/15/7/0/0						
**LEDD**	621.7	417.0	540.00	490.00	129	514.4	311.7	420.00	378.6	131	*p* = 0.059	
**BDI-I (/63)**	10.68	8.46	9.00	9.00	140	6.48	5.47	5.00	6.50	139	*p* < 0.001	**
**SAS (/42)**	15.05	5.84	15.00	7.00	131	12.33	5.59	12.00	7.00	137	*p* < 0.001	**
**FAQ (/30)**	3.78	6.01	1.00	5.00	124	1.12	2.29	0	1.00	125	*p* < 0.001	**
**PDQ-39 (%)**	28.19	17.83	25.96	22.44	130	15.12	11.49	13.46	14.10	138	*p* < 0.001	**
**Short IQCODE (/5)**	3.14	0.51	3.09	0.33	120	3.05	0.38	3.00	0.19	128	*p* = 0.039	*

Demographic and clinical data for FOG+ and FOG–. Both groups were matched for sex, age and disease duration.SD, standard deviation; IQR, Inter Quartile Range; FOG+, freezers; FOG–, non-freezers; M, male; F, female; R, right-handed; L, left-handed; A, ambidextrous; NA, not available; N, sample size; GBA, glucocerebrosidase gene mutation; MDS-UPDRS, Movement Disorder Society - Unified Parkinson’s Disease Rating Scale; LEDD, Levodopa Equivalent Daily Dose; BDI, Beck Depression Inventory; SAS, Starkstein Apathy Scale; FAQ, Functional Activity Questionnaire; PDQ-39, Parkinson’s Disease Questionnaire 39-item; IQCODE, short Informant Questionnaire on Cognitive Decline in the Elderly.* Significant at the unadjusted 5% level (value of *p* ≤ 0.05) (two-tailed); ** Significant at the Bonferroni-adjusted 5% level (value of *p* ≤ 0.05/16) (two-tailed).

Our outcome variable of interest, CUPRO-IS1, was significantly lower in the FOG^+^ compared to FOG^−^ (*u* = 12,651, *p* < 0.001). FOG^+^ presented significantly lower MoCA total scores (*u* = 12264.5, *p* = 0.007), as well as significantly higher TMT-A and TMT-B time scores (*u* = 8047.5, *p* = 0.021; *u* = 7,089, *p* < 0.001 respectively) and Delta TMT (*u* = 7,135, *p* < 0.001) compared to FOG^−^. No significant differences were observed for the CUPRO-IS2. Differences in neuropsychological measures between the two groups can be found in Table 2. No significant correlation was observed between the CUPRO scores and the FOG severity (MDS-UPDRS 2.13 /3.11). Results on the Spearman Correlations for the CUPRO scores in the FOG^+^ group can be found in Table 3.

**Table 2:** Differences in neuropsychological measures between FOG+ (*N* = 144) and FOG- (*N* = 144) matched on age, sex and disease duration.

	Variable	FOG+(*N* = 144)				FOG-(*N* = 144)				*N* FOG+ /FOG-	Value of *p*s	Significance
		*Mean*	*SD*	*Median*	*IQR*	*Mean*	*SD*	*Median*	*IQR*			
**CUPRO Evaluation System**	**IS**_**1**_ **(/3)**	1.81	1.17	2.00	2.00	2.28	1.03	3.00	1.00	144/144	*p* < 0.001	*
	**IS**_**2**_ **(/3)**	2.14	1.01	3.00	2.00	2.38	1.16	3.00	1.00	144/144	*p* = 0.084	
	**CUPRO total score (/6)**	3.94	2.10	4.00	4.00	4.66	1.78	6.00	2.00	144/144	*p* = 0.004	*
**Global Cognition**	**MOCA total score (/30)**	25.85	2.68	26.00	4.00	26.71	2.36	27.00	3.25	144/144	*p* = 0.007	*
**Psychomotor speed / Mental flexibility**	**TMT-A (sec)**	56.64	32.28	47.50	29.25	49.25	23.56	45.00	22.00	140/137	*p* = 0.021	*
	**TMT-B (sec)**	149.4	80.82	125.00	106.20	115.1	62.99	97.00	51.00	140/137	*p* < 0.001	*
	**Delta TMT (sec)**	92.79	71.66	74.50	94.25	65.80	49.60	48.00	46.00	140/137	*p* < 0.001	*

Neuropsychological assessment. The extended evaluation system: the first intermediate score (IS1) (our outcome variable of interest) evaluates the drawing procedure. The second intermediate score (IS2) evaluates visuo-constructive functions.N, sample size; SD, standard deviations; FOG+, freezers, FOG–, non-freezers; CUPRO, Cube drawing procedure, extended evaluation system of the Cube Copying Task; IS, intermediate score; TMT, Trail-Making-Test; Delta TMT: (TMT-B)–(TMT-A); MoCA, Montreal Cognitive Assessment.* Significant at the 5% level (*p*-value ≤ 0.05) (two-tailed).

**Table 3:** Spearman Correlations for the CUPRO scores in the FOG^+^ group.

	MDS-UPDRS II.13		MDS-UPDRS III.11		MDS-UPDRS II.13 + III.11	
	Coefficient *R*	*P*-values	Coefficient *R*	*P*-values	Coefficient *R*	*P*-values
**IS**_**1**_	0.04	0.7	−0.03	0.7	0.01	0.9
**IS**_**2**_	0.06	0.4	−0.04	0.6	0.03	0.7
**IS**_**1**_ **+ IS**_**2**_	0.07	0.4	−0.04	0.6	0.03	0.8
**Delta TMT**	0.08	0.4	−0.06	0.5	0.02	0.8
**MoCA total**	−0.04	0.6	0.07	0.4	−0.01	0.9

Spearman correlations.IS1, intermediate Score 1; IS2, intermediate Score 2; MDS-UPDRS, Movement Disorder Society - Unified Parkinson’s Disease Rating Scale; Delta TMT: (TMT-B)–(TMT-A); MoCA, Montreal Cognitive Assessment.

Given that depression can have an important impact on cognition, we repeated our analyses by additionally controlling for depression. After controlling additionally for this variable, our main outcome variable CUPRO-IS1 (*u* = 8,211, *p* = 0.010), as well as the variables for the TMT (TMTa: *u* = 5123.5, *p* = 0.006; TMTb: *u* = 5099.5, *p* = 0.005; Delta TMT: *u* = 5,418, *p* = 0.030) remained significantly lower in FOG^+^. Findings on the additional analyses can be found in Supplementary Tables S1, S2.

## 4 Discussion

The present study aimed to investigate if Freezing of gait (FOG) in Parkinson’s disease (PD) is associated with impairment in retrograde procedural memory. For this purpose, we used the CUPRO assessment tool (Pauly et al., 2022) and compared performance in FOG^+^ and FOG^−^, matched on age, sex and disease duration. The present study demonstrates significantly lower scores representing the performance in retrograde procedural memory in FOG^+^, even when matched for age, sex and disease duration to the control group. Similar observations on procedural learning have been described in FOG^+^ (Vandenbossche et al., 2013a).

Furthermore, we tested for differences in other cognitive domains. Previous studies have suggested cognitive deficits in FOG+ (Vandenbossche et al., 2013a; Cohen et al., 2014; Jha et al., 2015; Heremans et al., 2016), but only a few found no differences in cognition (Morris et al., 2020). These discrepancies can be explained by the varying definitions of FOG and non-controlled covariates. Our results support previous studies which demonstrated impaired psychomotor speed, procedural skills (Vandenbossche et al., 2013a; Heremans et al., 2016) and executive functions (Amboni et al., 2007; Peterson et al., 2016), such as mental flexibility in FOG^+^ compared to FOG^−^. We did not see any significant differences for visuo-constructive functions. Nevertheless, we need to keep in mind that a small test battery was used. To validate the findings, future research should apply a larger neuropsychological test battery.

Our findings of impaired procedural memory and mental flexibility, part of the executive functions, support the Vandenbossche model (Vandenbossche et al., 2013b). The model hypothesizes that those two functions, regulated by the frontostriatal circuitry, are crucial for understanding the pathogenesis of FOG. In case of disturbances of automaticity/procedural memory, one would expect a shift in neural activation from sub-cortical to cortical brain areas as a compensation strategy. In case of additional impairment of executive functions, this could lead to a FOG episode (Vandenbossche et al., 2013b). Recent brain imaging studies support this finding by describing increased involvement of attention as a compensatory strategy in PD compared to control subjects after motor learning (Wu et al., 2015). Functional neuroimaging studies suggested that FOG in PD is caused by abnormal interactions between frontoparietal cortical and subcortical structures, such as the striatum (Shine et al., 2013). This is in line with our observation of impaired retrograde procedural memory in FOG^+^, as the basal ganglia, especially the dorsolateral striatum, play an essential role in procedural memory (Mishkin and Appenzeller, 1987).

Measured by the absence of significant correlations, neither, global cognition, mental flexibility, nor retrograde procedural memory, were affected more severely by the worsening of the FOG symptoms. This previously mentioned shift might therefore not be gradual, defined by a temporal gradient but more by a spontaneous shift.

Furthermore, our findings that FOG^+^ show significantly more non-motor and motor symptoms, lower quality of life and higher disease stages compared to their matched control group, are in line with previous findings (Perez-Lloret et al., 2014). Given that depression can have an important impact on cognition, we repeated our analyses by additionally controlling for depression. The results for the main outcome variable, retrograde procedural memory, remained significantly lower in FOG^+^. This is in line with our observations made in a previous study comparing retrograde procedural memory in PwPD and control subjects, where we did not find significant associations between depression symptomatology and retrograde procedural memory (Pauly et al., 2022).

We took into consideration recently published recommendations for studies on cognition and FOG in PD (Monaghan et al., 2023); First, we ensured that the FOG cohorts were well characterized for clinical demographics including age, sex, education and, what is often neglected, for disease duration. Second, we reported medication status, by calculating the Levodopa Equivalent Daily Dose (LEDD) for each participant, so that the impact of dopamine medication can be interpreted. In the current study, we did not see any significant difference in LEDD between both groups. Third, apart from the novel CUPRO evaluation system, we used validated neuropsychological assessment tools and questionnaires to facilitate future comparisons across studies. The present study has the advantage that we included people with current and initial FOG symptoms. Given that dopaminergic medication can have a positive impact on gait abnormalities (Giladi, 2008), a medically well-adjusted patient may have his FOG masked.

Although the differences in dopaminergic medication were not statistically significant, they may still influence our outcome variables, as dopaminergic treatments can potentially shape the neural connectivity of cognitive networks in PD (Aracil-Bolaños et al., 2021). Since no significant correlation between retrograde procedural memory and LEDD have been observed in our previous study (Pauly et al., 2022), we do not anticipate a substantial impact on retrograde procedural memory. Furthermore, despite the fact that the years of education did not significantly differ between both groups, we cannot rule out the possibility that it might have an impact on our outcome variable, considering that previous findings have shown that the number of years of education completed is positively correlated with their cognitive functions (Lövdén et al., 2020).

Even though FOG is one of the main causes of falls and reduced quality of life, knowledge of treatment options, especially for non-invasive therapeutic approaches, is limited. Therefore, getting a deeper understanding of the relation between the pathophysiology of FOG and cognitive functions such as retrograde procedural memory is important, as these insights can lead to new hypotheses on the etiology of FOG. Previous findings demonstrated that cognitive rehabilitative training improves FOG symptoms in PwPD, leading to neuroplastic changes by reinforcing cognitive strategies (Walton et al., 2018). Research developing cognitive rehabilitation training reinforcing cognitive compensation strategies in people with FOG may have the potential to improve the quality of life of FOG patients.

The present study aimed to investigate if Freezing of Gait (FOG) in Parkinson’s disease (PD) is associated with impairment in retrograde procedural memory. By comparing retrograde procedural memory performance in FOG^+^ and FOG^−^, measured by the CUPRO assessment, we observed significantly lower CUPRO-IS1 scores, suggestive of impaired retrograde procedural memory, in FOG^+^, even when accounting for possible confounding factors such as age, sex, disease duration or depression.

Although FOG is a significant contributor to falls and a decline in the quality of life, our knowledge of treatment options, particularly non-invasive therapeutic methods, is still limited. Therefore, gaining insights into specific patterns of cognitive impairment, such as procedural memory in PwPD and FOG, and its suggested relationships with other cognitive domains in other studies, may improve our understanding of FOG’s causes. Consequently, a more thorough understanding of the cognitive deficits observed in PD may facilitate the targeted development of cognitive rehabilitation training and reeducation therapies. These efforts aim to preserve or even enhance cognitive function, ultimately leading to an improvement in the quality of life for individuals with PD.

## Data availability statement

The original contributions presented in the study are included in the article/Supplementary material, further inquiries can be directed to the corresponding authors.

## Ethics statement

All participants taking part in the Luxembourg Parkinson’s Study agreed and signed a written informed consent. The study was conducted in accordance with the local legislation and institutional requirements and has obtained a positive opinion from the National Research Ethics Committee (CNER Ref: 201407/13).

## Author contributions

LP: Conceptualization, Data curation, Formal analysis, Investigation, Methodology, Project administration, Visualization, Writing – original draft, Writing – review & editing. CP: Conceptualization, Data curation, Investigation, Methodology, Project administration, Writing – review & editing. MH: Conceptualization, Data curation, Investigation, Methodology, Project administration, Writing – review & editing. VS: Conceptualization, Data curation, Writing – review & editing. AR: Conceptualization, Data curation, Formal analysis, Investigation, Writing – review & editing. AL: Conceptualization, Data curation, Formal analysis, Investigation, Methodology, Project administration, Supervision, Writing – review & editing. RK: Conceptualization, Data curation, Formal analysis, Funding acquisition, Investigation, Methodology, Project administration, Resources, Supervision, Writing – review & editing.
