# Supplementary material for: Retrograde procedural memory is impaired in people with Parkinson’s disease with freezing of gait
Source: Front Aging Neurosci. 2024 Jan 5;15:1296323. doi: 10.3389/fnagi.2023.1296323 (PMC10797621; doi:10.3389/fnagi.2023.1296323)
Supplement: Supplementary file 1 [file Data_Sheet_1.pdf]

Supplementary Material

Retrograde Procedural Memory is impaired in people with Parkinson's Disease with Freezing of Gait

Laure PAULY*[1,2,3,4], Claire PAULY[1,3,4], Maxime HANSEN[1,3,4], Valerie E. SCHRÖDER[3,4], Armin RAUSCHENBERGER[3], Anja K. LEIST[5], Rejko KRÜGER*[1,3,4] on behalf of the NCER-PD Consortium

[1]Transversal Translational Medicine, Luxembourg Institute of Health, Strassen, Luxembourg;

[2]Faculty of Science, Technology and Medicine, University of Luxembourg, Esch-sur-Alzette, Luxembourg;

[3]Luxembourg Centre for Systems Biomedicine, University of Luxembourg, Esch-sur-Alzette, Luxembourg;

[4]Parkinson Research Clinic, Centre Hospitalier de Luxembourg, Strassen, Luxembourg;

[5]Department of Social Sciences, Institute for Research on Socio-Economic Inequality, University of Luxembourg, Esch-sur-Alzette, Luxembourg.

***Correspondence:** Laure Pauly, Luxembourg Institute of Health, 1A-B rue Thomas Edison, L-1445 Strassen, Luxembourg. Tel./Fax: +352 44 11 48 48; E-mail: laure.pauly@lih.lu; Rejko Krüger, Luxembourg Institute of Health, 1A-B rue Thomas Edison, L-1445 Strassen, Luxembourg. Tel./Fax: +352 44 11 48 48; E-mail: rejko.krueger@lih.lu

Figure 1 Supp.: Flowchart

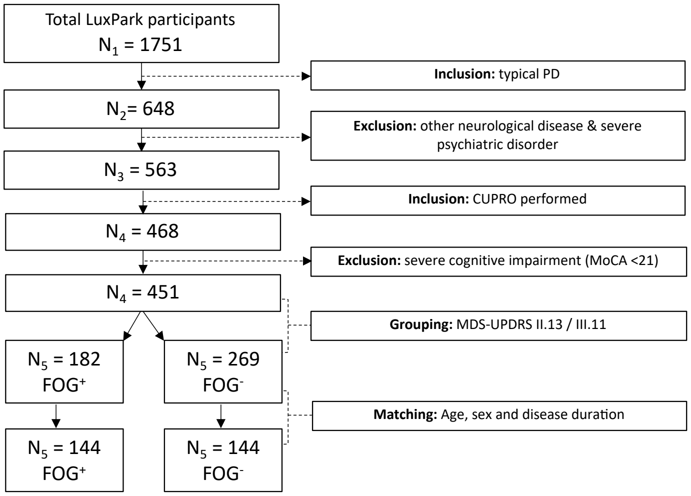

FOG+: Freezers; FOG-: non-Freezers; N: sample size; PD: Parkinson's Disease; CUPRO: Cube drawing PROcedure, extended evaluation system of the Cube Copying Task; MoCA: Montreal Cognitive Assessment; MDS-UPDRS: Movement Disorder Society - Unified Parkinson's Disease Rating Scale; MoCA: Montreal Cognitive Assessment

Table 1 Supp.: Demographic and clinical data for FOG+ (N = 118) and FOG- (N= 118) matched on age, sex, disease duration **and depression**.

Variable	Descriptive statistics										P-Values	Significance
	FOG+					FOG-					FOG+ vs. FOG-	
**N Total**	118					118						
**N GBA+ / GBA-**	18 / 98+2NA					9 / 94+15NA					p = 0.188	
**Sex, M / F**	86 / 32					83 / 35					p = 0.773	
	Mean	*SD*	*Median*	*IQR*	*N*	*Mean*	*SD*	*Median*	*IQR*	*N*		
**Age,** in years	67.53	10.35	69.12	12.82	118	66.75	9.94	67.53	13.49	118	p = 0.271	
**Disease duration,** in years	4.66	3.98	4.00	4.75	118	4.94	4.15	4.00	5.00	118	p = 0.493	
**Education,** in years	13.33	3.71	13.00	4.00	118	14.43	3.79	14.00	5.00	118	p = 0.017	*
**Languages spoken**	2.98	1.04	3.00	2.00	118	2.76	1.15	3.00	2.00	118	p = 0.527	
**MDS-UPDRS I (/52)**	10.03	5.98	9.00	7.00	118	8.45	5.12	8.00	6.00	117	p = 0.067	
**MDS-UPDRS II (/52)**	12.24	7.03	11.00	9.00	118	7.80	5.21	7.00	7.00	117	p < 0.001	**
**MDS-UPDRS III (/132)**	37.72	12.94	36.00	16.25	116	32.25	12.80	32.00	19.00	118	p = 0.002	**
**Modified Hoehn and Yahr**	2.26	0.52	2.00	0.50	117	1.96	0.45	2.00	0.00	118	p < 0.001	**
Stage 1 / 1.5 / 2 / 2.5 / 3 / 4 / 5	2/2/72/26/10/5+1NA					12/10/78/12/6/0						
**LEDD**	631.4	420.8	536.50	487.50	106	531.9	331.6	450.0	381.1	103	p = 0.129	
**BDI-I (/63)**	8.09	5.86	7.50	6.75	118	7.92	5.61	7.00	6.00	118	p = 0.905	
**SAS (/42)**	13.83	5.18	14.00	8.00	109	12.82	5.11	12.50	6.00	114	p = 0.134	
**FAQ (/30)**	3.10	5.32	1.00	4.00	102	1.33	2.35	0.00	2.00	104	p = 0.020	*
**PDQ-39 (%)**	24.92	17.08	21.79	22.12	107	17.63	11.91	16.03	14.74	113	p = 0.002	**
**Short IQCODE (/5)**	3.06	0.46	3.00	0.25	100	3.08	0.34	3.00	0.19	106	p = 0.554	

Demographic and clinical data for FOG+ and FOG-. Both groups were matched for sex, age, disease duration and depression. SD: Standard Deviation; IQR: InterQuartile Range FOG+: Freezers; FOG-: non-Freezers; M: Male; F: Female; R: Right-handed; L: Left-handed; A: Ambidextrous; NA: not available; N: sample size; GBA: glucocerebrosidase gene mutation; MDS-UPDRS: Movement Disorder Society - Unified Parkinson's Disease Rating Scale; LEDD: Levodopa Equivalent Daily Dose; BDI: Beck Depression Inventory; SAS: Starkstein Apathy Scale; FAQ: Functional Activity Questionnaire; PDQ-39: Parkinson's disease questionnaire 39-item; IQCODE: Short Informant Questionnaire on Cognitive Decline in the Elderly. * Significant at the unadjusted 5% level (p-value ≤ 0.05) (two-tailed); ** Significant at the Bonferroni-adjusted 5% level (p-value ≤ 0.05/16) (two-tailed).

Table 2 Suppl.: Differences in neuropsychological measures between FOG+ (N = 118) and FOG- (N = 118) matched on age, sex, disease duration **and depression.**

	Variable	FOG+				FOG-				N FOG+ /FOG-	P-Values	Significance
		Mean	*SD*	*Median*	*IQR*	*Mean*	*SD*	*Median*	*IQR*			
**CUPRO Evaluation System**	**IS1 (/3)**	1.85	1.16	2.00	2.00	2.22	1.08	3.00	1.75	118/118	p = 0.010	*
	**IS2 (/3)**	2.30	1.10	3.00	1.00	2.43	0.94	3.00	1.00	118/118	p = 0.583	
	**CUPRO total score (/6)**	4.14	2.00	4.00	3.00	4.65	1.78	6.00	2.00	118/118	p = 0.043	*
**Global Cognition**	**MOCA total score (/30)**	26.30	2.48	27.00	3.00	26.68	2.28	27.00	3.75	118/118	p = 0.305	
**Psychomotor speed / Mental flexibility**	**TMT-A (sec)**	59.05	40.30	48.00	15.00	46.75	18.64	43.00	25.00	115/113	p = 0.006	*
	**TMT-B (sec)**	136.7	75.02	110.00	97.50	109.10	56.73	95.00	46.00	115/113	p = 0.005	*
	**Delta TMT (sec)**	77.63	71.52	62.00	73.50	62.37	46.20	46.00	42.00	115/113	p = 0.030	*

Neuropsychological assessment. The extended evaluation system: the first intermediate score (IS_1) (our outcome variable of interest) evaluates the drawing procedure. The second intermediate score (IS_2) evaluates visuo-constructive functions. SD: Standard Deviations; N: Sample size; FOG+: Freezers; FOG-: non-Freezers; CUPRO: Cube drawing PROcedure, extended evaluation system of the Cube Copying Task; IS: Intermediate Score; TMT: Trail-Making-Test; Delta TMT: (TMT-B)–(TMT-A); MoCA: Montreal Cognitive Assessment.* Significant at the 5% level (p-value ≤ 0.05) (two-tailed).
